# Epidemiology of Influenza A Virus among Black-headed Gulls, the Netherlands, 2006–2010

**DOI:** 10.3201/eid2001.130984

**Published:** 2014-01

**Authors:** Josanne H. Verhagen, Frank Majoor, Pascal Lexmond, Oanh Vuong, Giny Kasemir, Date Lutterop, Albert D.M.E. Osterhaus, Ron A.M. Fouchier, Thijs Kuiken

**Affiliations:** Erasmus Medical Center, Rotterdam, the Netherlands (J.H. Verhagen, P. Lexmond, O. Vuong, A.D.M.E. Osterhaus, R.A.M. Fouchier, T. Kuiken);; Sovon Dutch Centre for Field Ornithology, Nijmegen, the Netherlands (F. Majoor);; Natuurmonumenten Dutch Association for Nature Conservation and Management, 's-Graveland, the Netherlands (G. Kasemir, D. Lutterop)

**Keywords:** influenza A virus, viruses, influenza, avian influenza, Charadriiformes, epidemics, virulence, disease reservoirs, zoonoses, black-headed gulls, the Netherlands

## Abstract

We sampled 7,511 black-headed gulls for influenza virus in the Netherlands during 2006–2010 and found that subtypes H13 and H16 caused annual epidemics in fledglings on colony sites. Our findings validate targeted surveillance of wild waterbirds and clarify underlying factors for influenza virus emergence in other species.

Wild waterbirds of the orders Anseriformes (ducks, geese, swans) and Charadriiformes (gulls, terns, shore birds) are the ultimate source of influenza A viruses for domestic birds and mammals, including humans (*1*). Knowledge of the epidemiology of these avian influenza viruses (AIVs) among wild waterbirds is necessary to improve surveillance and better clarify underlying factors in host-switching of AIV. Epidemiology of AIV in wild waterbirds has been studied mainly among ducks (order Anseriformes) (*2*) but is poorly known among gulls, despite their abundance and close association with humans (*3*). Therefore, we studied the epidemiology of AIV in one of the most common gull species in western Europe, the black-headed gull (*Chroicocephalus ridibundus*).

## The Study

Black-headed gulls (n = 7,511) were sampled year-round at multiple locations in the Netherlands during 2006–2010. Birds were captured by hand, leg-noose, or clap net; then, we determined their sex and age (first-year [FY] bird: nestling, fledgling; after first-year [AFY] bird) and weighed them. During the breeding season (April–July), 2,839 FY and 524 AFY birds were sampled at colony breeding sites. Three breeding sites were monitored annually during 2008–2010: Griend, De Kreupel, and Veluwemeer. At Griend, BHGU breeding success was also measured and used to compare breeding chronology to timing of infection (Technical Appendix). Outside the breeding season, 1,200 FY and 2,948 AFY birds were sampled in meadows and cities. Cloacal and oropharyngeal swab samples were collected from each bird and tested for AIV by using matrix (M)–specific reverse transcription PCR (RT-PCR) and, if positive, for H5 and H7 subtypes by using hemagglutinin (HA)–specific RT-PCR. Virus culture was attempted on all M RT-PCR–positive samples by egg inoculation. Virus isolates were classified to HA subtype by hemagglutination inhibition assay and to neuraminidase (NA) subtype by using RT-PCR (*4*,*5*). Blood samples were collected from an arbitrary subset of 134 FY and 214 AFY birds and tested for anti-AIV antibody by nucleoprotein (NP)–specific ELISA (*6*). Statistics were performed by using software RStudio version 0.95.265 (www.r-project.org). Additional analyses on AIV prevalence among male versus female birds, dead versus live birds, recaptured birds, and capture bias were performed (online Technical Appendix).

**Figure 1:**
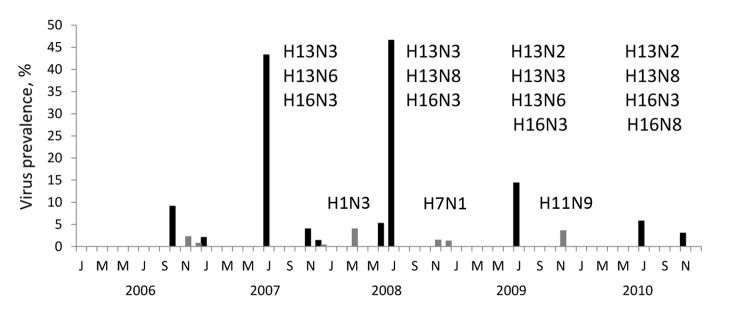
Avian influenza virus prevalence among 7,511 black-headed gulls, the Netherlands, 2006–2010. Cloacal and oropharyngeal samples were collected once from each gull for virus detection. Influenza virus subtypes detected are shown above virus positives. Bars indicate virus prevalence (No. PCR-positive samples/no. gulls sampled per month). Black bars represent gulls in their first year (FY) of life, comprising nestling and fledgling stages; gray bars represent after-first year (AFY) gulls.

**Figure 2:**
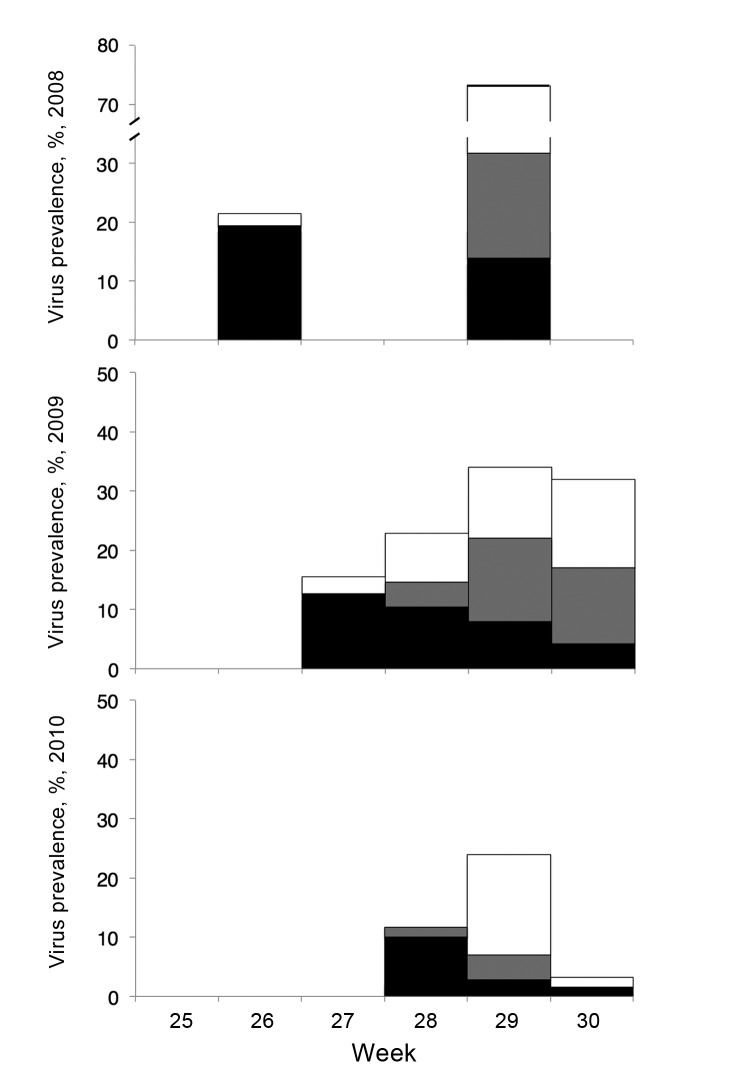
Avian influenza virus prevalence and hemagglutinin subtype (H) distribution of 871 first-year black-headed gulls sampled on the colony site of Griend during 2008–2010. Bars indicate virus prevalence (no. PCR-positive samples/no. sampled per week). Black bar sections, H13; gray bar sections, H16; white bar sections, unknown H subtype.

Our results showed that AIV epidemics in black-headed gulls occurred annually during June and July, with a peak monthly prevalence of 47% during 2008 (Figure 1, Table 1). These epidemics were detected in FY birds only and were limited to subtypes H13 and H16; subtype H13 and H16 viruses represented 100% of all virus isolates and 55% of RT-PCR positive birds. In contrast, no AIVs were detected in 524 AFY birds sampled during the breeding season. Annual epidemics were detected in 2 of 3 colonies sampled annually during 2008–2010 (Technical Appendix Table 1). More detailed investigation on Griend showed that, although H13 and H16 viruses were detected each year, H13 was the only (2008, 2009) or predominant (2010) subtype detected on the first day of virus detection of each breeding season (Figure 2, Table 2). In 2008 and 2009, H16 was detected the next sampling day, which was 1–2 weeks later. H16 was or became the predominant subtype during 2008–2010; H13 prevalence decreased during that period. The source of H13 and H16 viruses causing these epidemics is unknown. Possible sources are breeding or nonbreeding BHGU, other gull species at the colony sites, and freshwater ponds (if present) at the colony sites. Nonbreeding BHGU tend to wander among colony sites. BHGU that breed north of the Netherlands arrive in the Netherlands from July 1 onwards (F. Majoor, unpub. data).

**Table 1:** Number of black-headed gulls sampled per month for detection of avian influenza virus among 7,511 black-headed gulls, the Netherlands, 2006–2010*

	No. sampled											
Y, Age	Jan	Feb	Mar	Apr	May	Jun	Jul	Aug	Sep	Oct	Nov	Dec
2006												
FY	0	0	0	0	6	365	0	0	0	11	74	70
AFY	0	0	0	0	1	0	0	0	1	7	90	138
2007												
FY	96	28	1	0	0	167	37	0	1	6	100	73
AFY	72	39	0	1	34	2	0	1	1	4	153	275
2008												
FY	11	32	33	0	1	632	290	0	0	4	47	108
AFY	37	61	75	0	33	9	42	0	1	5	68	160
2009												
FY	169	43	0	0	0	295	383	0	0	0	45	57
AFY	740	172	3	0	31	82	55	0	0	0	56	288
2010												
FY	60	52	2	0	0	212	451	3	0	2	33	39
AFY	232	135	11	0	45	128	61	7	1	4	40	71
*FY, gulls in their first year of life, comprising nestling and fledgling stages; AFY, after first year.												

**Table 2:** Number of 871 FY black-headed gulls sampled per week, Griend, the Netherlands, June–July of 2008–2010*

Month and week	No. samples		
	2008	2009	2010
June			
25	0	46	44
26	98	70	33
27	0	71	74
July			
28	0	48	60
29	101	50	71
30	0	47	62

*FY, gulls in their first year of life, comprising nestling and fledgling stages.

Results from Griend also showed that these epidemics occurred after onset of fledging. The first detection of AIV on Griend (during the last week of June 2008, the first week July 2009, and mid-July 2010) occurred 1–3 weeks after onset of fledging. Also, of 871 FY birds, AIV was detected only in FY birds with an average length of <200 mm, above which they are considered to be fledged (*7*). Possible explanations for timing of the epidemic could be increased mobility after fledging and, therefore, increased contact rate; access to water, facilitating more efficient virus transmission; and increased susceptibility of fledglings as a result of immature body condition and loss of maternal antibodies.

Body condition did not differ notably between virus-positive and virus-negative FY birds sampled on the same day (p>0.05, Mann-Whitney Wilcoxon test), except for during the third week of July during 2009 (p = 0.046) and 2010 (p = 0.0004), when virus-positive birds had lower body condition. This suggests that, overall, H13 and H16 virus infections are nonpathogenic for BHGU. Previous studies found no clinical signs (*8*) or histological lesions (*9*) in gulls naturally infected with H13 or H16 virus. No notable differences in virus prevalence were found related to gender, no consistent differences in virus prevalence were found related to capture method, and no AIVs in dead BHGU were detected outside epidemics (Technical Appendix).

Outside the breeding season, AIV prevalence was much lower, and no H13 or H16 viruses were isolated; AIV were exclusively isolated from AFY birds and were typed as H1N3, H7N1, and H11N9 (Figure 1, Table 1). Additionally, a single H5 virus was detected by using H5 RT-PCR in an AFY gull sampled in December 2006. H13 viruses have been isolated from ring-billed gulls (*Larus delawarensis*) outside the breeding season (*10*). The lack of detection of H13 and H16 viruses in BHGU outside the breeding season in our study provides no support for virus circulation at low prevalence in overwintering FY birds. Our sample size of FY birds sampled outside the breeding season (n = 1,200) may be around the theoretical limit to detect the presence of these viruses in the population, assuming a virus prevalence of 0.5% in a homogeneously distributed population (*11*). However, a nonhomogeneous BHGU population structure outside the breeding season might support a situation in which susceptible FY gulls are present year-round and thus facilitate the circulation of AIV throughout the year at an even lower prevalence.

Prevalence of anti-AIV antibodies detected in FY birds sampled outside the breeding season was statistically more significant (15/59 [25.4%]) than in FY birds sampled during the breeding season (4/75 [5.3%]) (p<0.01, Fisher exact test). The 4 seropositive FY birds were fledglings (n = 55); nestlings (n = 20) were seronegative. There was no statistically significant difference in the seropositivity of AFY gulls sampled during (40/101 [39.6%]) and outside (45/113 [39.8%]) the breeding season (p>0.05, Fisher exact test). These results suggest that FY birds during the breeding season are the most susceptible category to become infected with AIV.

## Conclusions

We describe annual AIV epidemics in BHGU colonies. These epidemics were caused by AIV subtypes H13 and H16 and occurred in FY birds during the second half of the breeding season, with prevalence rates of up to 72% per week. On most sampling days, infected and noninfected FY birds had similar body conditions, suggesting H13 and H16 viruses are nonpathogenic for BHGU. These findings broaden our view on AIV dynamics in populations of gull species often closely associated with humans and facilitate more targeted sampling of colonial nesting waterbirds. Further research is needed to show if the same AIV dynamics apply to other gull species and other geographic areas and to clarify the epidemiology of AIV in wild birds and factors that influence emergence of influenza in domestic animals and humans.

Technical Appendix Descriptions of sampled sites and sampling of black-headed gulls.
