# Supplementary material for: Epidemiology of Influenza A Virus among Black-headed Gulls, the Netherlands, 2006–2010
Source: Emerg Infect Dis. 2014 Jan;20(1):138–41. doi: 10.3201/eid2001.130984 (PMC3884729; doi:10.3201/eid2001.130984)
Supplement: Technical Appendix — Descriptions of sampled sites and sampling of black-headed gulls. [file 13-0984-Techapp-s1.pdf]

Article DOI: http://dx.doi.org/10.3201/eid2001.130984

Epidemiology of Influenza A Virus in Black-headed Gulls, the Netherlands

Technical Appendix

Sampled Sites

Location and Characteristics of Black-headed Gull (BHGU) Colony Sites

Three breeding colony sites were intensively monitored for AIV: Griend, De Kreupel and Veluwemeer. The island of Griend (lat-y 53.252, long-x 5.254) is home to the largest BHGU colony of the Netherlands, consisting of approximately 30,000 pairs (*1*). The colony size was estimated at 35,166 breeding pairs in 2008, 32,780 in 2009, and 31,408 in 2010 (*2-4*). This human-uninhabited and protected island is located in the Wadden Sea. We sampled BHGU on Griend for AIV from 2008 to 2010. De Kreupel consists of a chain of small islands (lat-y 52.797, long-x 5.229) in the IJsselmeer, and is home to the second-largest BHGU colony of the Netherlands, consisting in 2010 of 9231 breeding pairs (*5*). We sampled BHGU on De Kreupel from 2007 to 2010. Veluwemeer (lat-y 52.399, long-x 5.711) is an inland site with three small islands that are home to a BHGU colony of approximately 1400 pairs (*5*). We sampled BHGU at Veluwemeer for AIV from 2006 to 2010.

Other bird species than BHGU also use these three sites for breeding. Sandwich Tern (*Thalasseus sandvicensis*), Common Tern (*Sterna hirundo*), Arctic Tern (*Sterna paradisaea*) and Oystercatcher (*Haematopus ostralegus*) breed on Griend (*4*). Common Tern (*Sterna hirundo*), Tufted Duck (*Aythya fuligula*), Gadwall (*Anas strepera*) and Mallard (*Anas platyrhynchos*) breed on De Kreupel and on the islands of Veluwemeer. Great Cormorant (*Phalacrocorax carbo*) breeds on De Kreupel, and Egyptian Goose *(Alopochen aegyptiaca)* and Mute Swan (*Cygnus olor*) breed on the islands of Veluwemeer. In addition, these colony sites are used as stop-over sites for migrating shorebirds, Griend

and De Kreupel more so than the more inland located Veluwemeer (F.Majoor, unpublished data). Vegetation on the colony site of Griend consists of grasses (e.g. *Ammophila arenaria*, *Elymus arenarius*) and nettles (*Urtica dioica*). Vegetation on De Kreupel and the islands of Veluwemeer consists mainly of fireweed (*Chamerion angustifolium*) and nettles.

In addition to the colony sites of Griend, De Kreupel and Veluwemeer, BHGU were sampled on other colony sites within the Netherlands for the presence of AIV in the summer of 2008. These colony sites were located in the Wadden Sea on the island of Ameland (lat-y 53.435, long-x 5.640, 3650 pairs in 2010 [*5*]), near Bargerveen (lat-y 52.681, long-x 7.032, 829 pairs in 2008 [*6*]), Blauwestad (lat-y 53.171, long-x 7.012, 1500 pairs in 2009 [*7*]), Tjeukemeer (lat-y 52.888, long-x 5.800, 1500 pairs, F.Majoor, unpublished data) and Zoetermeer (lat-y 52.075, long-x 4.532, 500 pairs, F.Majoor, unpublished data). BHGU found dead were sampled at the colony sites listed above, and at Houtribdijk (lat-y 52.626, long-x 5.423), Arnhem (lat-y 51.985, long-x 5.910) and Hilversum (lat-y 52.229, long-x 5.167).

Distinction between coastal and inland colony sites was described previously (*8*) based on location of the colony site and food collected by BHGU breeding there. Remarkably, breeding at coastal colony sites (Griend, Ameland, De Kreupel and Blauwestad) started 1-2 weeks later than at more inland located colony sites (Veluwemeer, Bargerveen and Tjeukemeer) (F.Majoor, unpublished data).

Measurement of BHGU Breeding Success

The BHGU colony on Griend has been monitored for breeding success since 1964 (*1*). For this purpose, breeding success enclosures (fences surrounding multiple nests) have been used on Griend since 1994 to monitor nests from egg laying until fledging of the chicks. Between 2008 and 2010, guards of the island monitored three (2008 and 2009) or four (2010) enclosures, which contained on average 7.9 nests (minimum 2, maximum 16). Enclosures 1 to 3 were located on lat-y 53.25232, long-x 5.25131; lat-y 53.25205, long-x 5.25117 and lat-y 53.25205, long-x 5.25104, respectively. Enclosure 4, used in 2010, was adjacent to enclosure 3. Enclosures surrounding multiple nests were visited ≥1 per week to monitor nests from egg laying until fledging of the chicks. Within the enclosures the number of hatchlings per day was estimated based on disappearance of the egg and presence of a chick without a leg ring that subsequently was ringed the same day. Within the enclosures, wing length of all ringed nestlings was monitored. FY gulls were considered fledglings the first day the wing length was ≥ 200mm.

Sampled Black-headed Gulls

Ethical Approval

An independent Animal Ethics Committee of the Erasmus Medical Center (Stichting DEC Consult) approved this study (permit numbers 122-07-09, 122-08-12, 122-09-20, 122-10-20).

Geographical Origin of BHGU

Sampled BHGU were first-year (FY) and after-first-year (AFY) birds of mixed origin, one part originating from breeding colonies in the Netherlands and the other part originating from breeding colonies north/north-east of the Netherlands in Scandinavia, Poland or the Baltic States (*9*). Based on sightings, AFY gulls that breed north/north-east of the Netherlands leave the Netherlands by April 1st at the latest to migrate to their breeding colony sites. They may be observed again in the Netherlands from July 1st onwards, followed 1-2 weeks later by FY gulls from those breeding colony sites (F. Majoor, unpublished data).

Determination of Sex, Age and Body Condition of BHGU

Birds were sexed based on total head-bill length and bill depth when captured after fledging (*10*). Birds were aged based on total head-bill length when captured before fledging (*11*) and based on plumage when captured after fledging (*12*). In addition, FY birds were categorized based on wing length as nestling (<200 mm) or fledgling (≥200 mm) (*11*). To calculate a scaled mass index of body condition (called body condition), body mass and head-bill length as length value were used (*13*).

Additional Analyses

Analysis of Gender Differences

Gender was determined for 4,356 of 7,511 sampled BHGU (58.0%). Of these 4356 gulls, 317 (7.3%) were FY birds sampled within the breeding season. Of 4,356 birds, 1,149 birds were female (26.3%) and 3207 of 4,356 were male (73.6%), suggesting there is a capture bias towards male gulls in the current dataset. Among FY gulls of which the gender was known, 3 of 290 female (1.0%) and 7 of 874 male (0.8%) gulls were M RT-PCR positive. Of AFY gulls, 5 of 859 (0.6%) female gulls and 7 of 2,326 male gulls (0.3%) were M RT-PCR positive. No statistically significant difference in AIV prevalence between genders among FY ($p>0.05$, Fisher's exact) and AFY birds ($p>0.05$, Fisher's exact) was observed.

Comparison of Live and Dead Birds

Next to sampling BHGU alive, 158 samples from the cloaca and oropharynx were collected from 113 FY and 45 AFY gulls found dead from 2007 to 2010. In dead FY gulls, viruses were detected in June and July of 2008 on three colony sites (Technical Appendix Table 2). When H13 and H16 viruses were detected in dead FY birds, viruses of the same HA subtypes were detected in FY gulls sampled alive at the same date and location (Additional analyses - Table S2). Samples from cloaca and oropharynx collected on Griend on the day that AIV were detected in multiple dead FY gulls (July 14th, 2008) suggest that more virus was present in dead FY gulls (mean cycle-threshold (Ct) value of 27, SD = 4.7, n = 20) than in live FY gulls (mean Ct value of 32, SD = 4.5, n = 73) ($p=0.00009$, Mann-Whitney Wilcoxon). No viruses were detected in 45

AFY gulls that were found dead in April (n=1) and June (n=17) of 2007, in January (n=1), February (n=1), April (n=3), May (n=2), June (n=10), July (n=2), October (n=1) and November (n=2) of 2008, in January (n=1) and June (n=1) of 2009 and in July (n=3) of 2010.

Analysis of Recaptured Gulls

FY gulls that were captured and sampled more than once on the colony site of Griend within the breeding season were used to investigate whether an individual FY gull was infected multiple times with AIV within a single breeding season. On the colony site of Griend, 2, 14 and 17 FY gulls were captured and sampled twice within the breeding season of 2008, 2009 or 2010, respectively (Technical Appendix Table 3). AIV were detected in 7 of the 33 recaptured FY gulls. Of the seven FY gulls that tested AIV positive, six were detected positive once and one was detected positive twice with an interval of 1 week. Of the FY gull that tested AIV positive twice, the first time H16N3 AIV was isolated, one week later the sample was M RT-PCR positive, but no virus could be isolated and characterized. Therefore, serial infections with one subtype or cross-infections of H13 and/or H16 viruses within a single host and season could not be demonstrated.

In addition to these 33 FY gulls that were captured and sampled twice on the colony site of Griend, 215 gulls were captured and sampled twice at other locations in the Netherlands between 2006 and 2010; none of them tested positive for AIV. In addition, ten gulls were sampled three times; again, none of them tested positive for AIV.

Analysis of Capture Method

To determine whether the decrease in body condition during the course of the breeding season was associated with the capture method used, we compared body condition—as well as wing length and virus prevalence—between birds caught by hand and birds caught by clap net, on 3 days

in 2009 and 2010 when methods were used in parallel (Technical Appendix Table 4). Capture method was not associated with a consistent change in body condition. In contrast, wing length of FY birds captured by hand (n=126) was significantly lower than wing length of FY birds captured by clap net (n=65) on 3 out of 3 days ($p<0.01$, Mann-Whitney Wilcoxon test). Virus prevalence of FY birds captured by hand did not differ from virus prevalence of FY birds captured using a clap net on 2 out of 3 days. However, on 1 day (July 21st, 2010), birds captured by hand (n=39) had significantly lower wing length ($p<0.05$, Mann-Whitney Wilcoxon test), the same mean body condition and significantly higher virus prevalence ($p<0.05$, Mann-Whitney Wilcoxon test) than birds captured using a clap net (n=32).

References

1. Van Dijk K, Oosterhuis R. Origin and trends in numbers and breeding success of Black-headed Gulls *Larus ridibundus* breeding on Griend (Wadden Sea). Limosa. 2010(83):21–35[cited 2013 Nov 21]. http://nou.nu/limosa/limosa_samenvatting.php?language=UK&nr=4157

2. Lutterop D, Kasemir G. Griend vogels en bewaking 2008. 's-Graveland: Vereniging Natuurmonumenten; 2009 [cited 2013 Nov 21]. http://www.natuurmonumenten.nl/sites/default/files/Griend%20Vogels%20en%20Bewaking%202008.pdf

3. Lutterop D, Kasemir G. Griend vogels en bewaking 2009. 's-Graveland: Vereniging Natuurmonumenten; 2010 [cited 2013 Nov 21]. http://www.natuurmonumenten.nl/sites/default/files/Griend%20Vogels%20en%20Bewaking%20Deel%201%20-%202009.pdf

4. Lutterop D, Kasemir G. Griend vogels en bewaking 2010. 's-Graveland: Vereniging Natuurmonumenten; 2011 [cited 2013 Nov 21]. http://www.natuurmonumenten.nl/sites/default/files/Griend%20Vogels%20en%20Bewaking%20-%20Deel%201.pdf

5. Boele A, Van Bruggen J, Van Dijk AJ, Hustings F, Vergeer JW, Ballering L, et al. Broedvogels in Nederland in 2010. Nijmegen: SOVON Vogelonderzoek Nederland; 2012.

6. Van Dijk AJ, Boele A, Hustings F, Koffijberg K, Plate CL. Broedvogels in Nederland in 2008. Beek-Ubbergen: SOVON Vogelonderzoek Nederland; 2010.

7. Boele A, Van Bruggen J, Van Dijk AJ, Hustings F, Vergeer JW, Plate CL. Broedvogels in Nederland in 2009. Nijmegen: SOVON Vogelonderzoek Nederland; 2011.

8. Stienen EWM, Arts FA, De Boer P, Beeren WJ, Majoor F. Broedresultaten van kokmeeuwen in Nederland in 1997. Sula. 1998;12:1–11.

9. Speek BJ, Speek G. Thieme's vogelatlas. Zutphen: Thieme; 1984.

10. Palomares LE, Arroyo BE, Marchamalo J, Sainz JJ, Voslamber B. Sex– and age-related biometric distribution of Black-headed Gulls *Larus ridibundus* in Western European populations. Bird Study. 1997;44:310–7. http://dx.doi.org/10.1080/00063659709461066</jrn>

11. Van Dijk JGB, Stienen EWM, Gerritsen S, Majoor FA. Reproductie van de Kokmeeuw in kust- en binnenlandkolonies. Limosa. 2009(82):13-22 [cited 2013 Nov 21]. http://www.boerenlandvogels.nl/sites/default/files/Limosa_82-1-2009_13-22_Majoor.pdf

12. Grant PJ. Gulls: a guide to identification. Arrington, USA: Buteo books; 1986.

13. Peig J, Green AJ. New perspectives for estimating body condition from mass/length data: the scaled mass index as an alternative method. Oikos. 2009;118:1883–91 [cited 2013 Nov 21]. http://dx.doi.org/10.1111/j.1600-0706.2009.17643.x

Technical Appendix Table 1. Avian influenza virus prevalence in first-year Black-headed Gulls sampled at 3 breeding colony sites (Griend, De Kreupel, Veluwemeer) in the Netherlands during the 2006–2010 breeding seasons*†

	Griend					De Kreupel					Veluwemeer				
Year	Date	No. sampled	No. PCR positive	No. VI positives	No. each subtype	Date	No. sampled	No. PCR positive	No. VI positive	No. each subtype	Date	No. sampled	No. PCR positives	No. VI positives	No. each subtype
2006	ND	ND	ND	ND	ND	ND	ND	ND	ND	ND	6 May	6	0	0	NA
	ND	ND	ND	ND	ND	ND	ND	ND	ND	ND	4 Jun	199	0	0	NA
	ND	ND	ND	ND	ND	ND	ND	ND	ND	ND	12 Jun	94	0	0	NA
	ND	ND	ND	ND	ND	ND	ND	ND	ND	ND	23 Jun	72	0	0	NA
2007	ND	ND	ND	ND	ND	5 Jul	36	16	10	H13N3 (1)<br>H13N6 (3)<br>H16N3 (6)	ND	ND	ND	ND	ND
	ND	ND	ND	ND	ND	ND	ND	ND	ND	ND	ND	ND	ND	ND	ND
	ND	ND	ND	ND	ND	ND	ND	ND	ND	ND	10 Jun	144	0	0	NA
	ND	ND	ND	ND	ND	ND	ND	ND	ND	ND	ND	ND	ND	ND	ND
	ND	ND	ND	ND	ND	ND	ND	ND	ND	ND	30 Jun	23	0	0	NA
2008	28 Jun	98	20	19	H13N8 (19)	9 Jun	64	0	0	NA	7 May	1	0	0	NA
	14 Jul	100	72	32	H13N8 (14)<br>H16N3 (18)	24 Jun	77	0	0	NA	6 Jun	126	0	0	NA
	ND	ND	ND	ND	ND	15 Jul	78	23	9	H13N8 (5)<br>H16N3 (4)	25 Jun	18	0	0	NA
2009	17 Jun	46	0	0	NA	15 Jun	47	0	0	NA	ND	ND	ND	ND	ND
	24 Jun	70	0	0	NA	26 Jun	64	0	0	NA	1 Jul	29	0	0	NA
	2 Jul	71	11	9	H13N2 (9)	29 Jun	66	0	0	NA	ND	ND	ND	ND	ND
	9 Jul	48	11	7	H13N2 (4)<br>H13N6 (1)<br>H16N3 (2)	3 Jul	63	0	0	NA	ND	ND	ND	ND	ND
	15 Jul	50	17	11	H13N2 (3)<br>H13N3 (1)<br>H16N3 (7)	6 Jul	50	0	0	NA	ND	ND	ND	ND	ND
	22 Jul	47	15	8	H13N2 (1)<br>H13N6 (1)<br>H16N3 (6)	13 Jul	26	1	1	H13N2 (1)	ND	ND	ND	ND	ND
2010	23 Jun	44	0	0	NA	24 Jun	63	0	0	NA	ND	ND	ND	ND	ND
	30 Jun	33	0	0	NA	2 Jul	43	0	0	NA	5 Jun	30	0	0	NA
	7 Jul	73	0	0	NA	8 Jul	60	0	0	NA	4 Jul	41	0	0	NA
	14 Jul	60	7	7	H13N2 (1)<br>H13N8 (5)<br>H16N8 (1)	19 Jul	39	0	0	NA	ND	ND	ND	ND	ND
	21 Jul	71	17	5	H13N2 (1)<br>H13N8 (1)<br>H16N3 (3)	ND	ND	ND	ND	ND	ND	ND	ND	ND	ND

	Griend					De Kreupel					Veluwemeer				
Year	Date	No. sampled	No. PCR positive	No. VI positives	No. each subtype	Date	No. sampled	No. PCR positive	No. VI positive	No. each subtype	Date	No. sampled	No. PCR positives	No. VI positives	No. each subtype
	26 Jul	60	2	1	H13N2 (1)	ND	ND	ND	ND	ND	ND	ND	ND	ND	ND
Total		871	172	99			776	40	20			783	0	0	

*ND, no data; NA, not applicable.

†This sample (n=2,430) is a subset of the 7,511 BHGU sampled once for virus detection and shown in Figure 1, main manuscript. It excludes birds older than 1 year (n=3,472), First-year birds sampled outside the breeding season (n=1,200) and those sampled during the breeding season on colony sites other than the above 3 (n=410). Details of breeding colony sites are described in Technical Appendix Sampled Sites.

Technical Appendix Table 2**.** Avian influenza virus prevalence in first-year black-headed gulls found dead or captured alive in the Netherlands during 2007– 2010.

			Dead		Alive	
Year	Date	Location	No. PCR positive/no. sampled (%)	Subtype	No. PCR positive/no. sampled (%)	Subtype
2007	05 Jun	Houtribdijk	0/4 (0)	NA	0	NA
	06 Jun	Houtribdijk	0/1 (0)	NA	0	NA
2008	22 Jan	Arnhem	0/1 (0)	NA	0	NA
	06 Jun	Veluwemeer	0/3 (0)	NA	0/126 (0)	NA
	09 Jun	De Kreupel	0/3 (0)	NA	0/64 (0)	NA
	20 Jun	Bargerveen	0/4 (0)	NA	0/52 (0)	NA
	24 Jun	De Kreupel	0/10 (0)	NA	0/77 (0)	NA
	25 Jun	Veluwemeer	0/10 (0)	NA	0/18 (0)	NA
	28 Jun	Griend	0/15 (0)	NA	20/98 (20)	H13N8 (19)
	29 Jun	Blauwestad	1/6 (17)	H13N8 (1)	13/81 (16)	H13N8 (4)
	06 Jul	Ameland	1/5 (20)	H13N8 (1)	39/115 (34)	H13N3 (1); H13N8 (9); H16N3 (12)
	08 Jul	Houtribdijk	0/1 (0)	NA	0/6 (0)	-
	14 Jul	Griend	20/30 (67)	H13N8 (4); H16N3 (5); H16N8 (2)	73/101 (72)	H13N8 (14); H16N3 (18)
	15 Jul	De Kreupel	0/7 (0)	NA	23/78 (29)	H13N8 (5); H16N3 (4)
	03 Dec	Hilversum	0/1 (0)	NA	0/5 (0)	NA
2009	31 Jan	Arnhem	0/1 (0)	NA	0	NA
	26 Jun	De Kreupel	0/1 (0)	NA	0/64 (0)	NA
	29 Jun	De Kreupel	0/2 (0)	NA	0/66 (0)	NA
	03 Jul	De Kreupel	0/5 (0)	NA	0/63 (0)	NA
2010	20 Jan	Hilversum	0/1 (0)	NA	0	NA
	03 Feb	Hilversum	0/1 (0)	NA	0/1 (0)	NA
	19 Jul	De Kreupel	0/1 (0)	NA	0/39 (0)	NA

*NA, not applicable.

Technical Appendix Table 3. Detection of avian influenza virus in avian influenza virus-positive first-year black-headed gulls that were recaptured and sampled within this study period on the colony site of Griend, the Netherlands

FY bird no.	Capture sequence	Sample date	Result PCR	Wing length (mm)	Head-bill length (mm)	Body mass (g)	SMI
1	1	28-Jun-08	–	172	66,8	176	208
	2	14-Jul-08	+	233	71,9	202	206
2	1	28-Jun-08	–	181	70,7	240	228
	2	14-Jul-08	+	259	77,8	218	170
3	1	02-Jul-09	–	236	76,1	273	247
	2	09-Jul-09	+	275	79	234	181
4	1	09-Jul-09	–	251	70	204	248
	2	15-Jul-09	+	265	71	199	234
5	1	14-Jul-10	+	281	79,1	246	199
	2	21-Jul-10	+	289	79,7	167	141
6	1	07-Jul-10	–	260	73,4	193	205
	2	21-Jul-10	+	278	74,5	155	166
7	1	21-Jul-10	+	286	77,1	204	194
	2	26-Jul-10	–	288	77,3	210	221

SMI, scaled mass index of body condition.

Technical Appendix Table 4. Avian influenza virus prevalence, wing length, and SMI of body condition of first-year Black-headed Gulls captured on Griend on the same sampling day*

			Capture technique			Significance of difference between hand-captured and clap-net-captured birds	
. Year	Date of sampling		Per sampling day	Hand	Clap net	p-value	Test
2008	28-Jun	Mean wing length	201	201	-	NA	NA
		Mean SMI	216	216	-	NA	NA
		No. virus positive/No. sampled (%)	20/98 (20)	20/98 (20)	-	NA	NA
	14-Jul	Mean wing length	232	232	-	NA	NA
		Mean SMI	196	196	-	NA	NA
		No. virus positive/No. sampled (%)	72/100 (72)	72/100 (72)	-	NA	NA
2009	17-Jun	Mean wing length	169	169	-	NA	NA
		Mean SMI	237	237	-	NA	NA
		No. virus positive/No. sampled (%)	0/46 (0)	0/46 (0)	-	NA	NA
	24-Jun	Mean wing length	202	202	-	NA	NA
		Mean SMI	247	247	-	NA	NA
		No. virus positive/No. sampled (%)	0/70 (0)	0/70 (0)	-	NA	NA
	2-Jul	Mean wing length	229	229	-	NA	NA
		Mean SMI	238	238	-	NA	NA
		No. virus positive/No. sampled (%)	11/71 (15)	11/71 (15)	-	NA	NA
	9-Jul	Mean wing length	244	244	-	NA	NA
		Mean SMI	204	204	-	NA	NA
		No. virus positive/No. sampled (%)	11/48 (23)	11/48 (23)	-	NA	NA
	15-Jul	Mean wing length	253	252	301	NA	NA
		Mean SMI	195	196	170	NA	NA
		No. virus positive/No. sampled (%)	17/50 (34)	17/49 (35)	0/1 (0)	NA	NA
	22-Jul	Mean wing length	272	267	281	p<0.01	Mann-Whitney Wilcoxon
		Mean SMI	188	182	199	p<0.05	Mann-Whitney Wilcoxon
		No. virus positive/No. sampled (%)	15/47 (32)	10/31 (32)	5/16 (31)	p=1	Fisher's exact
2010	23 Jun	Mean wing length	183	183	-	NA	NA
		Mean SMI	217	217	-	NA	NA
		No. virus positive/No. sampled (%)	0/44 (0)	0/44 (0)	-	NA	NA
	30-Jun	Mean wing length	211	211	-	NA	NA
		Mean SMI	246	246	-	NA	NA
		No. virus positive/No. sampled (%)	0/33 (0)	0/33 (0)	-	NA	NA
	7-Jul	Mean wing length	247	239	272	p<0.01	Mann-Whitney Wilcoxon
		Mean SMI	240	247	220	p<0.01	Mann-Whitney Wilcoxon
		No. virus positive/No. sampled (%)	0/73 (0)	0/56 (0)	0/17 (0)	p=1	Fisher's exact
	14-Jul	Mean wing length	247	247	-	NA	NA
		Mean SMI	208	208	-	NA	NA
		No. virus positive/No. sampled (%)	7/60 (12)	7/60 (12)	-	NA	NA
	21-Jul	Mean wing length	266	247	283	p<0.01	Mann-

						Whitney Wilcoxon
	Mean SMI	186	183	190	p>0.05	Mann-Whitney Wilcoxon
	No. virus positive/No. sampled (%)	17/71 (24)	17/39 (44)	0/32 (0)	p<0.01	Fisher's exact
26-Jul	Mean wing length	287	-	287	NA	NA
	Mean SMI	206	-	206	NA	NA
	No. virus positive/No. sampled (%)	2/60 (3)	-	2/60 (3)	NA	NA

*SMI, scaled mass index; NA, not applicable.
